# Exploring AI-Driven Feedback as a Cultural Tool: A Cultural-Historical Perspective on Design of AI Environments to Support Students’ Writing Process

**DOI:** 10.1007/s12124-025-09894-8

**Published:** 2025-02-01

**Authors:** Irina Engeness, Siv M. Gamlem

**Affiliations:** 1 Department of Languages, Literature and Culture, Faculty of Teacher Education and Languages, Østfold University College, Halden, Norway; 2 Faculty of Humanities and Education, Institute of Pedagogy, Volda University College, Volda, Norway

**Keywords:** Cultural-historical theory, Galperin, Feedback, Assessment for learning, Artificial intelligence

## Abstract

This study draws on the cultural-historical perspectives of Vygotsky and Galperin to examine the role of AI-generated feedback within the Assessment for Learning (AfL) process in fostering students’ development as learners. By leveraging Galperin’s concept of cultural tools and the developmental role of human activity, elaborated in his dissertation written almost a hundred years ago, this study elucidates how this theoretical framework can enhance our understanding of the pedagogical value of individually tailored feedback from AI, ultimately contributing to human development and inspire the Design Principles (DPs) of AI-based educational technologies. Essay Assessment Technology (EAT), designed according to the suggested DPs, is presented to illustrate the application of AfL strategies in schools, highlighting its potential to enhance students’ learning and their development as learners.

## Introduction

In our constantly changing world, the main goal of schooling is to support students in learning how to go about learning (Engeness, 2020; Smith et al., 2016). By fostering learning-to-learn strategies, we empower students to become life-long learners, capable of navigating the complexities of an ever-evolving landscape. Such an approach ensures that they not only acquire knowledge but also develop the ability to continually expand and apply their understanding in diverse and dynamic contexts. Educational researchers and practitioners have emphasised the importance of developing life-long learning strategies and efforts have been made to enhance critical thinking, problem-solving skills, and capacities in learning to learn among students. For example, Inquiry-Based Science Education emphasises learning through scientific inquiry, encouraging students to engage in hands-on activities and real-world problem-solving. This approach promotes the development of critical thinking and self-directed learning (García-Carmona, 2020; Linn & Eylon, 2011). Constructivist Learning Theory suggests that learners construct their own understanding based on experiences and reflections. This theory supports a student-centred approach where learners develop their understanding actively by engaging in collaborative activities, dialogue, and continuous construction of knowledge. Such an environment may foster the ability to learn how to learn, as students become active participants in their educational journey (Weinstein et al., 2018). Research indicates that integrating formative assessment can significantly enhance students’ self-regulated learning (SRL) (Pintrich & Zusho, 2002; Zimmerman, 2008) and improve their academic outcomes. Brandmo et al. (2020) argue that combining these approaches fosters students’ ability to manage their own learning and call for increased research and collaboration between educators and researchers to effectively implement these practices. By merging formative assessment with SRL, instructional practices can be improved, resulting in better educational experiences and achievements for students.

However, November 2022 marked the beginning of significant advancements in the field of Artificial Intelligence (AI) which raised concerns about student’ learning (Baron, 2023). The innovations within language technologies and machine learning have significantly altered the educational landscape; they are widely regarded as a turning point for educational methodologies (Holmes & Tuomi, 2022) and are identified as revolutionary tools that indicate a paradigm shift in education (Gentile et al., 2023; Guan et al., 2020; Huang et al., 2021). Broadly speaking, AI can be described as ‘machine-based systems that can, given a set of human-defined objectives, make predictions, recommendations, or decisions that influence real or virtual environments. AI systems interact with us and act on our environment, either directly or indirectly. Often, they appear to operate autonomously and can adapt their behaviour by learning about the context’ (UNICEF, 2021, p.16).

Although the emergence of AI in education holds a significant promise, it remains a novel area requiring extensive research to fully uncover its pedagogical potential, such as feedback provided by AI in students’ writing process and the pedagogical design of AI technologies. Many of these technologies are produced for primarily commercial purposes which also imposes challenges for their pedagogical use. To reveal the pedagogical potential and enhance design of AI for educational purposes, it is essential to offer a robust conceptualisation of feedback provided by AI and discuss its link with the design of AI technologies. We believe that cultural-historical theory may provide a valuable framework to enhance our understanding the pedagogical value of AI technologies. Prior to presenting the legacies of the cultural-historical scholars, Vygotsky and Galperin, this study discusses the role of feedback in the learning process, highlighting both opportunities and challenges associated with AI-driven feedback.

## The Role of Feedback in the Writing Process

Feedback can be defined as “information that is given to the learner about his or her performance on a learning task, usually with the objective of improving the performance” (Ur, 1996, p. 242). Meanwhile, written corrective feedback (WCF) refers to “… any feedback provided to a learner, from any source, that contains evidence of learner mistake of language form” (Russell & Spada, 2006, p. 134). Feedback requires three aspects of information to be effective: (1) the learner’s prevailing performance in relation to the intended goal, (2) the intended level of performance; and (3) the solution to bridge the prevailing and intended performance levels (Black & Wiliam, 1998; Sadler, 1989; Wiliam & Leahy, 2007). As such, corrective feedback is thought to be beneficial for second language acquisition as it allows students to pick up grammatical features that may be lost due to the discontinued access to learning standards (Ellis, 2009). Research into the most effective form of WCF is important for teachers who spend an inordinate amount of time on providing such feedback and for learners who wish to improve their grammatical accuracy (Hopfenbeck et al., 2023; Storch, 2018).

Several researchers identify core principles and strategies of feedback to support learning: (i) feedback must be an integral part of the teaching and learning process (Andrade, 2010; Black & Wiliam, 2009; Gamlem & Smith, 2013), (ii) be understandable to the receiver (Black & Wiliam, 1998; Gamlem, 2014; Sadler, 1998) and (iii) the students must be allowed to act on the feedback (refine, revise and practice) (Gamlem & Smith, 2013; Sadler, 1998). Furthermore, feedback should be linked with learning goals (Hattie & Timperley, 2007; Gamlem & Smith, 2013; Sadler, 1989), and should cause thinking (Black & Wiliam, 2009; Butler & Winne, 1995; Nicol, 2021). In doing so, feedback may foster students’ self-regulating capacity (Brandmo et al., 2020).

Feedback for learning and understanding has been formulated by Hattie and Timperley (2007) as a three-stage process where students should be posing three questions and given answers to by the help of external sources: *Where am I going* (the goals), *Where am I* (current stage), and, *Where am I going next* (the process)? In this three-stage process four major levels of feedback should be aimed at to support learning. The levels are feedback about the task, the processing of the task, self-regulation, and the self. The level at which feedback is directed influences its effectiveness for learning. While feedback on the self-level (which involves personal evaluations of an individual, as student’s character traits) is found to be the least effective, feedback on the process- and self-regulation level are powerful in terms of mastery of the learning process, and feedback on task level is powerful when the task is useful for improving strategy processing or enhancing self-regulation (Hattie & Timperley, 2007). The feedback about the process provides opportunities to close the gap between potential and performance (Sadler, 1989). Feedback loops should be tailored according to the ability of the system to react to the feedback, and the shortest (and most powerful) feedback loops are those that take place in daily classroom interactions between student(s) and teacher and are integrated in the learning processes (Wiliam & Leahy, 2007). These feedback loops can provide opportunities for students to elaborate on what is not yet understood, investigate misconceptions, and engage students in deep learning (Black & Wiliam, 2009; Gamlem & Munthe, 2014). Metacognitive approaches to instruction have shown to increase the degree to which students will transfer to situations without the need for explicit prompting since they become more self-regulated (Andrade, 2010; Butler & Winne, 1995).

There is a need to pay attention to the students’ writing processes, revision of work, and uptake of feedback (Carless & Boud, 2018). That is, to how learners process and respond to the feedback provided, or to the impact of this feedback on subsequent writing. Such strategies have been described as Assessment for Learning (AfL) approach with the aim to improve formative assessment practices in the classroom by developing distinct criteria to clarify how to reach curriculum goals (Hopfenbeck et al., 2012). Engaging in AfL practices enables students to develop essential skills for effective learning, particularly the ability to learn how to learn, which is crucial in the 21st century (Engeness, 2021a; Engeness et al., 2021; Smith et al., 2016). Consequently, the effectiveness of teachers’ AfL practices is critical for fostering students’ learning and their development as learners (Gamlem, 2015). Additionally, modern learning environments urge that both teachers and students become proficient in utilising AI tools to enhance educational practices in classrooms.

## AI-Driven Feedback: A New Promise?

Early AI is described as automatic essay assessment (AEA) (Dikli, 2006; Landauer et al., 2003) and the studies on AEA have been aligned with the developments in text mining and natural language processing (NLP). Recently, automated written feedback (AWF), has been increasingly recognised for its utility in language education. This technology is becoming a preferred aid in language learning and teaching due to its effectiveness in providing feedback (Burstein et al., 2020; Ding & Zou, 2024; Li, 2021; Ranalli, & Yamashita 2022). AWF is utilised for formative assessment within teaching settings, offering targeted feedback that supports learning (Stevenson & Phakiti, 2014; Weigle, 2013). AWF systems use AI, particularly natural language processing (NLP) algorithms, to evaluate and provide feedback on written texts. Systems like Criterion and Pigai are among the examples of this technology. Similar to AWF are Automated Corrective Feedback (ACF) tools in language learning (Shadiev & Feng, 2023). The benefits of ACF tools include immediate, tailored feedback that supports diverse language skills and promotes independent learning (Rahimi et al., 2024). However, challenges include ensuring feedback accuracy and relevance and the potential reduction in human interaction which is vital for contextual and motivational aspects of learning. The emergence of generative AI technologies like ChatGPT, Microsoft Copilot, Gemini and others is expected to enhance ACF tools by providing more accurate and comprehensive feedback (Barrot, 2023). Former studies have found that the quality in students’ text improve with the use of AI-generated feedback (Engeness & Mørch, 2016; Mørch et al., 2017). However, there are also some indications that automated feedback may position learners to be reliant on the provided feedback, and thus decreasing self-initiated corrections (Asiri & Khadawardi, 2024; Baron, 2023). Hopfenbeck et al. (2023) discuss both the potential benefits and challenges of using AI to support student learning. AI can significantly enhance formative assessment by providing personalised feedback, automating assessment tasks, and supporting large-scale, sustainable assessment practices. It can deliver timely and high-quality feedback, thereby improving the overall formative assessment process. However, there are challenges to consider, such as the necessity for teacher professional development in AI literacy, ethical concerns, and the risk of biases in AI algorithms.

In summary, AWFs, ACFs and ChatGPT have been primarily used to support language learning with a focus on English as a target language (Shi, & Aryadoust, 2024) and the potential of AI to enhance students’ learning and their understanding of the learning process is yet to be revealed. In addition, the majority of studies on the role of student, teacher and AI feedback are based on empirical investigations and are lacking theoretical grounds. We believe that the cultural-historical perspective offers a powerful conceptualisation of cultural tools that may enhance our understanding of the pedagogical potential of feedback especially feedback from AI-powered technology to support an AfL approach. Such use of the cultural-historical theory is innovative, showcasing the richness and boundless potential of the theory developed in the 20th century to inspire and advance pedagogy in the 21st century and beyond.

## A Cultural-Historical Perspective

### The Role of Cultural Tools

A key concept in Vygotsky’s work is the idea of cultural tools as mediating means and a crucial connecting link between a subject (for example, a student) and an object (for example, what a student is going to learn) (Vygotsky, 1981). Vygotsky considered the characteristics and the quality of cultural tools to be of primary importance in students’ learning and development (Arievitch & Haenen, 2005; Stetsenko & Arievitch, 2002) by emphasising their psychological significance, for example, in his discussion about tools and signs (Engeness, 2021b; Vygotsky, 1980, 1981). Briefly, this discussion can be summarised as follows: through his analysis of Köhler’s studies on the intelligence of higher apes, Vygotsky investigated the distinctions in tool usage between animals and humans. He recognised that the role tools played in apes’ interactions with their surroundings differed significantly from their role in human activities (Vygotsky, 1997). Vygotsky argued that humans utilised both tangible and abstract tools, such as conceptual and linguistic devices (such as feedback), within the framework of social and practical activities, thereby bridging the gap between an individual and their environment. The argument continues that the use of tools as mediating means employed in practical activities leads to transformations in human consciousness. While employed in practical activities, in the process of mastering these *tools*, they gain their unique meanings for humans and are internalised as *signs*. For Vygotsky, the psychological importance of these tool-signs in the evolution of human consciousness distinguishes their use significantly from that of animals, highlighting a unique aspect of human development (Engeness, 2021b).

However, Vygotsky did not fully develop the concept of cultural tools and their role in human practical activities. A contemporary of Vygotsky, Galperin has greatly extended Vygotsky’s legacy though examining the difference between animal (manual) and human (psychological) tools. In his candidate dissertation entitled *Psychological Significance and Difference between Tools Use by Humans and Auxiliary Means by Animals*, defended in 1938, in Kharkiv Psycho-Neurological Academy, Galperin studied the differences in tool use between humans and animals and argued that there was a fundamental difference between the tools developed by humans and what he called auxiliary means (i.e., manual tools) used by animals (Galperin et al., 2023). Galperin posited that animals utilised tools in a spontaneous manner, which mirrored the instinctual nature of their actions. Animal tools lack the capability for preservation for future use and do not possess specific application domains. Their functions are not reflected in their form, they originate from nature, and they cannot be used within a social and collective production framework. Galperin concluded that characteristics of animal tools underscored their deficiencies and viewed them as intermediary connections between animals and their environment. These auxiliary means did not create avenues for novel activities; rather, it was the inherent and reflexive behaviour of the animals that dictated the application of these tools.

To consider the mediating role of the human tool, Galperin believed that it was not sufficient to examine the relationship between the tool and the purpose of the activity since this relationship showcased the tool’s technical and rational characteristics, such as its effectiveness in accomplishing the target task. Yet, neither the link to the objective nor the tool’s technical aspects provided a basis to uncover the tool’s role as a mediational mean. However, through examining *how a human employs the tool* and *its effectiveness to reach the intended goal*, the *mediational role of the tool* may be revealed. By employing this perspective in our case, the mediational role of feedback may be explored through examining students’ interactions and use of feedback in the writing process. Hence, to explore the dynamic between the tool and its mediational potential, it’s important to evaluate the tool’s (feedback) functions and how appropriate it is for fulfilling the intended purpose (write a text according to distinct criteria). Galperin believed that it was crucial to examine the use of the tool through the lens of a system of operations encapsulated in this tool. From this perspective, a tool could embody two distinct systems: (i) a system of mediated operations that emerged from social practices involving tool use and encapsulated in these tools – psychological tools, and (ii) a system of manual operations that evolved through the utilisation of a hand as a natural tool – manual tools.

By analysing the experiment that Galperin conducted (Galperin et al., 2023), he concluded that when a tool was integrated into the arm’s operational system, it ceased to have its unique functions and instead took on the role of an extended arm. In doing so, the tool did not introduce new capabilities to the user but merely provided a minor modification of the capabilities that already existed. Galperin defined such tools as *manual* that did not have any psychological significance for humans. However, when the arm adapted to become part of the operations mediated by a tool, it conformed to the demands of the tool and abandoned its natural functions. This adaptation allowed for novel forms of interaction with the surrounding world, bestowing the tool with its historical and *psychological importanc*e. Galperin offered an example of a child mastering the spoon:

First, a child attempts to hold the spoon as close as possible to its head. He puts his fingers and even his whole fist on to the spoon’s head. The purpose of such an attempt becomes obvious. Only when the nanny makes the child hold the handle of the spoon and together, they scoop up the porridge, the child with a sharp movement raises the spoon to his mouth. The spoon is not levelled and most porridge pours out; however, the child is moving in the same way as if he was bringing his fist to his mouth. Functionally, the spoon is no more than an extension of the child’s hand and the closer the child’s hand is to the spoon’s head, the more likely it is to enter the child’s mouth. The simple logic of a spoon as a tool is visible when having scooped some porridge, the bottom of the spoon is scratched against the edge of the plate. After that the filled spoon is lifted up vertically to the mouth’s level, to establish its horisontal position. Only after such a position has been established, that the child directs the spoon straight to his mouth….

A spoon in the child’s hand is a required substitution for a hand and, as such, a bad substitution. Only after a period of training, a child develops skills for using a spoon: not to move it immediately to the mouth, but at first to lift it up horisontally. For a long time, a child attempts to hold the spoon in his fist at the lowest part of the spoon’s handle. It is only when reaching the pre-school age, he masters how to use the spoon while complaining about the inconvenience of holding the spoon’s handle in his palm positioned upwards (a usual way to hold a spoon for adults) (Galperin et al., 2023, p. 7).

Consequently, tool-mediated operations involved not the arm’s movements as such, but its movements with the tool adjusting to the practices of using this tool determined by the target task. Based on these premises, a learner’s ability to identify these useful operations are crucial for performing a productive activity with the tool (feedback). In other words, feedback offered to students during their writing process, may gain its psychological significance when learners master the activities encapsulated in the feedback. In the discussion that follows, we consider the psychological significance of the feedback offered to the students by the AI-driven Essay Assessment Technology (EAT) in the writing process.

Galperin concluded that the use of tools by humans gave rise to a new reality. This reality was shaped by the characteristics of the tools and the historically and socially evolved practices of working with these tools. Thus, human psychological tools are not merely intermediary objects between the human and the environment (as animals’ means), but they encapsulate the operations and activities that can be conducted with these tools, embedded within a broader cultural context. Therefore, to fully understand the psychological significance of cultural tools, it is important to consider the role of the activities with these tools (Galperin et al., 2023).

### The Developmental Role of a Human Activity

When analysing the children engagement with the target tool in his research (the children of 2–8 years old were tasked to pull out the toys of different shapes and colours out of a deep rectangular box by using the spade – a blade attached to a stick at a right angle), Galperin et al. (2023) identified the four phases in the development of the activities aimed to master the tool-spade. During the initial phase, titled as *trial and error*, children experimented with high-speed movements with the spade, most of which were unsuccessful. The children struggled to handle the tool effectively and adjust based on how the tool could be used. Their actions were primarily manual and instinctive, reacting to different situations without much conscious thought. However, as time passed, the children used the successful ways of using the spade more frequently, leading to an overall improvement in how they used the tool. In the second phase, titled *monitoring the flow of activity*, the children performed the tasks, through numerous attempts, until they discovered an advantageous positioning for using the tool-spade with toys. Upon encountering these advantageous positions by chance, the children analysed the elements that led to these situations. This analysis divided the activity into two phases: ‘before’, where actions are accidental and unconscious, and ‘after’, where actions become intentional and conscious. In the third phase of *persistent interference*, children’s actions were seen as ‘good mistakes,’ as they proactively used strategies that were successful before to create beneficial situations. Unlike the initial stage where their actions were random, or the second phase where they passively hoped for advantageous moments, in the third phase, the children actively attempted to engineer these situations. Such attempts showed a progression from merely reacting to situations to using insights gained from past experiences to form new strategies. This phase was characterised by both action and reflection, where children did not only act but also thought through and evaluated their actions critically. In the fourth phase - *objective regulation*, children achieved mastery over the tasks they perform. This stage was marked by the children’s ability to smoothly control the progression of their activities, making quick adjustments whenever needed. This ability indicated that the children were guiding their actions through a conscious understanding of both the object they were interacting with and the tool they were using, considering the specific characteristics necessary for the task at hand. Essentially, this phase was defined by two key achievements: first, the children developed an awareness of the connections between the features of their environment and, second, they became adept at using tools, demonstrating refined skill in their movements. Such development represented a significant leap in children’s thinking, where actions were not just reactions to the environment but were informed by a deep understanding of the tool and the activity that employed this tool. Galperin showed that, a child’s thinking was initially visible as spontaneous external actions in response to changing circumstances gradually transformed into deliberate actions. This transformation illustrated how a child’s thinking was deeply intertwined with their interactions with the environment, moving from random, external actions to intentional, mindful behaviour. This progression not only highlighted the learning capacity of children but also underscored the significance of practical experience in shaping their thinking and understanding. Galperin concluded that the development of the children’s thinking was visible in their growing ability to select useful operations and adjust them to the conditions of the surrounding reality.

These findings have several implications for understanding the importance of learners’ engagement in practical activities with cultural tools. *First*, the relationship between intellectual and practical activities highlights their intrinsic connection. Initially, children’s intellectual activities arise by chance within practical interactions and are recognised and utilised deliberately in later stages. Over time, the interactions of a child undergo a transformation, evolving into psychological activity. This process illustrates how children gradually become aware of and intentionally apply the strategies they initially discovered accidentally, signifying a key progression in their thinking and cognitive development. *Second*, the dynamics between the operations and objects (tasks) evolve. At first, operations are employed inadvertently, but they gradually become influenced by the task’s demands and the child’s contemplation on how to interact with the target object. Through this process, the child recognises the link between the object and the operations employed, which in turn initiates changes in the object. Operations that were initially part of practical activities transform into mental operations that shape the nature of subsequent practical tasks. Thus, from a psychological perspective, the child’s thinking progresses beyond their practical activity, indicating a forward movement in cognitive development where mental planning and understanding precede and guide physical actions. *Third*, the evolution from practical to cognitive activities underscores the expansion of the child’s conscious grasp of these tasks. When children consciously comprehend an activity, they begin to apply this understanding in different situations, marking the transition from hands-on to cognitive activities and thus moving to the subsequent stage of mastering the tool. Through both successful and unsuccessful engagements with objects, the child starts to discern the relationships between objects and tools, facilitating their progression to the next stage.

Children’s consciousness develops during their practical activity by mastering various operations and gradually realising their content, opportunities and limitations (Galperin et al., 2023 p. 47).

Galperin concluded that higher psychological functions were nothing else but the mediated and meaningful actions of a person. The ability of a person to engage in meaningful actions reflected the advanced organisation of the human inner world and attitude to the external world. Galperin further conceptualised the significance of the learners’ engagement in practical activities with cultural tools for their development:

Human consciousness differed from animal consciousness not in its individual elements and not in its composition but in its organisation, which presented itself in relation to the external world and to reality. (Galperin et al., 2023, p. xl)

The significance of an action is determined by the context of the target task and the tools that are employed in this action. This implies that the value and purpose of an action are not inherent but are derived from its utility in solving a specific problem or achieving a particular goal, as well as from the appropriateness and effectiveness of the tools used in the process. The interaction between the task, the action, and the tool creates a framework within which the action gains its meaningfulness. Meaningful actions are developed through the development of meanings and in doing so, humans develop their understanding of the surrounding world. Galperin draws a powerful conclusion:

The development of meanings is nothing else but the process of the development of meaningful activities. This was the pathway of the development of freedom of human consciousness (Galperin et al., 2023, p.xl).

These arguments found their deeper exploration in Galperin’s research from the early 1950s on the development of human mental activity, particularly aiming to enhance students’ learning. Building upon Vygotsky’s notion that psychological functions first exist as external and social interactions before becoming internal and individual processes, Galperin examined how the internalisation of these functions signifies the transformation of thought from an external, interpersonal level to an internal, personal level. This concept of internalisation, coupled with the process of externalisation, was further refined by Galperin in the phases of the development of mental actions (Arievitch & Haenen, 2005; Engeness & Lund, 2020). Through his work, Galperin sought to elucidate how mental actions evolve, starting from their external manifestation in social interactions to their internalisation as individual cognitive processes. Galperin conceptualised the transformation of action from its external manifestation to internal mental processes as a gradual transformation, beginning with tangible, object-oriented activities and progressing towards entirely mental actions. This evolution occurs via a sequence of intermediary steps that include social communication and individual speech (Engeness & Lund, 2020; Gamlem, 2014). Galperin identified six distinct phases through which this transformation occurs: *motivation*,* orientation*,* materialised action*,* communicated thinking*,* dialogical thinking*,* and finally*,* acting mentally*. In the initial *motivational phase*, a learner’s attitude towards and relationship with the intended learning outcomes are established. The focus is on setting the stage for learning, creating interest, and recognising the value of what is to be learned. Following motivation, the learner enters the *orientation phase*, where they develop a comprehensive plan for action. This orienting basis is a generalised plan or approach that guides the subsequent actions of the learner, helping them to understand how to proceed. The overview of the entire process termed by Galperin as the *operational scheme of thinking* should be presented for the learners while creating an orientation, can be achieved through one of three strategies:

#### Incomplete

Here, learners discover through trial and error, leading to a slow learning process fraught with mistakes (as described in the experiment in Galperin’s dissertation). This method is particularly vulnerable to even minor changes in the learning environment.

#### Complete and Provided by the Teacher

In this type of orientation, learners receive comprehensive instructions from the teacher about the key aspects of what they are to learn and the approach they should take. This ensures learners have all the necessary tools and a clear plan for their learning activities.

#### Complete and Constructed by the Learners

This orientation involves learners identifying the characteristics of the target concept by themselves, however, by using a generalised approach offered by the teacher. By adopting this strategy, learners create an orientation tailored for a particular task. Galperin believed that this approach not only enhances learners’ understanding of the learning process but also fosters their development as autonomous and agentic learners. Learners move from *materialised action* to *communicated thinking* occurs as they engage with material or materialised objects (such as feedback) and begin to articulate their understanding of these objects through speech. During the materialised action phase, actions are outwardly focused, linking the learner with other learners and the broader external environment. This external focus serves as a bridge to the internal cognitive processes that follow.

The transition from communicated thinking to *dialogical thinking* involves learners replacing speech that is oriented towards the external world with an internal representation of that speech. In dialogical thinking, the direction of action turns inward, as learners engage in an internal dialogue, effectively communicating with themselves as if they were another person. This internalisation of dialogue marks a critical step in the development of independent thought, where learners not only absorb information from their surroundings but also critically engage with it internally, refining their cognitive abilities and fostering deeper understanding. The learner’s capacity to engage in dialogical thinking as a form of action showcases the journey that the action has taken from its initial, tangible (materialised) phase to its internalised, dialogical phase. In the final phase, the action transitions to being executed through hidden speech - acting mentally. This phase marks a significant development in the learning process, where the earlier practice of artificially breaking down the action into discrete steps is abandoned, allowing the action to unfold in its most fluid and natural form. It is during this phase that the action becomes highly automated, signifying that the learner has internalised the process to such an extent that it can be carried out with minimal conscious effort. This level of automation represents the pinnacle of mastery and learners’ profound understanding of the action.

In summary, Galperin’s conceptualising of human psychological tools urges the discussion about how these tools may acquire such a significance for humans. Galperin argues that this significance can be revealed in the context of carefully designed learning activities. The phases of the development of mental actions may inform such a design. Such an approach sheds lights on the importance to examine (i) learners’ interactions with the feedback from the AI-driven EAT and (ii) how the structure of the writing process enhances learners’ meaning-making of the feedback as a cultural tool that empowers their learning and development as learners.

## A Cultural-Historical Perspective on Feedback and Design of AI Environments to Support Students’ Writing Process

Drawing from Galperin’s research, feedback can be considered a cultural tool that bridges the gap between the learner and the external environment, encompassing the teacher, peers, and the criteria for writing. Utilising feedback effectively in the writing process can lead not only to improving students’ texts, but also to developing their understanding of a writing process. As students engage and develop their understanding of feedback it acquires unique meaning and becomes internalised as a sign, reflecting students’ ability to implement the feedback to improve their texts. The mediational role of feedback can be examined by analysing how students engage and use it in their writing. To thoroughly explore the link between feedback and its mediational potential, it is essential to evaluate its purpose, functions and effectiveness in achieving the desired outcomes, such as write a text according to specific criteria. From the cultural-historical perspective, different types of feedback encapsulate their essential characteristics. For example, Language feedback encompasses the feedback on grammar, spelling and punctuation; Content feedback – the subthemes covered in the texts and the subthemes that can be addressed in further drafts and Structure and Organisation feedback reflects the parts present in the text and the parts to be further included, whereas organisation feedback reflects the argument flow in the text. In addition, feedback as a cultural tool encapsulates operations of interactions with it. Therefore, it is crucial for learners to reveal and master these operations which should open new opportunities that would otherwise be inaccessible. Only then can feedback be considered a cultural tool with psychological significance for learners.

Galperin’s research on children mastering of activities, their gradual understanding of the activities and development of their consciousness showcases the importance of pedagogy, to design, structure and facilitate learning activities. It is argued that students’ growing understanding is deeply intertwined and affected by the environment which can be represented by a teacher, peers and the design of the learning activity or the writing process students engage in. Students’ productive writing process with feedback, can be summarised into several points.

*First*, students’ external interactions with the feedback, the teacher and the peers influence and are connected with the students’ writing process and the quality of their texts. Over time, their interactions undergo transformations and become students’ psychological activities. Such transformations signify the students’ developing understanding of their learning process.

*Second*, at the beginning, while mastering the writing process, students’ operations with feedback can be employed inadvertently, however, gradually, students enhance their understanding about the practices of interactions with feedback, it’s the role in the writing process and meeting the criteria of the target assignment. The link between the text and the received feedback becomes more evident, and such an understanding initiates changes students make in their texts. Operations that are performed initially as students’ external interactions with the feedback, the teacher and the peers transform into internal mental operations with the written text. In doing so, students’ thinking progresses beyond their practical activities and acquires the guiding function of their interactions with the text. To enhance students’ thinking, the pedagogical design of learning activities/writing process would seem to be crucial.

*Third*, the transition from practical to cognitive activities highlights the students’ growing understanding of the target task, the role of feedback and the writing process. As students develop a conscious comprehension of the writing process, they may begin to apply this knowledge in different situations, marking the shift from practical to mental engagements. Through both successful and unsuccessful initial interactions with feedback, the teacher and the peers, students begin to recognise the relationships between their quality of their texts and the interactions which enhances their understanding of the writing process.

Students’ interactions with feedback, both successful and unsuccessful, illuminate the necessity of explicitly revealing and teaching effective practices of engagement with feedback within a carefully structured learning process. Without such intentional pedagogical support, students are left to navigate learning through trial-and-error approaches, a method that, as Galperin’s work in the 1960s and 70s underscored, is neither efficient nor optimal for cognitive development (Galperin, 1966, 1968). Over the course of two decades of experimentation in schools, Galperin developed his phases of development of mental activities, highlighting the critical role of systematically designed instruction in fostering meaningful learning (Arievitch & Haenen, 2005; Engeness, 2021c; Podolsky, 2002;).

These insights underscore the importance of guiding students to move beyond trial-and-error interactions with feedback and towards deliberate, purposeful engagement that supports their learning trajectory. Drawing from Galperin’s research, feedback can be considered a cultural tool that bridges the gap between the learner and the external environment, encompassing the teacher, peers, and the criteria for writing.

To summarise, students’ understanding develops through practical interactions with feedback as they master operations encapsulated in the AI-offered feedback, gradually realising the content, opportunities, and limitations of these interactions. Students’ interactions between the assignment, the text, and the feedback forms a framework within which the action (writing process) acquires its meaningfulness. A meaningful writing process emerges through the development of meanings, enabling students to deepen their understanding of how to write a good text and, in a broader sense what it means to learn.

### Pedagogical Design Principles

Galperin’s legacy and specifically his phases of the development of mental activities have implications for outlining the pedagogical design principles (DP) of the AI-powered environments aimed to facilitate and offer feedback in the writing process. These DPs build up and extend our previous efforts on the design of digital learning environments to enhance students’ learning and their understanding of learning in digital environments (Engeness, 2021a).

#### DP1 Target Concept Alignment

when designing an AI technology aimed to offer individually tailored feedback in the writing process, it is important to identify (a) the target concept that the students need to grasp (what text the students are expected to write) and (b) the essential characteristics or structural components of that target concept (the essential characteristics of the texts which will be used as assessment criteria). The essential characteristics of the target concept will inform the type of feedback students receive during the writing process. As students work towards understanding the target concept, feedback will be tailored to address these fundamental elements, guiding them through the structural components that define the concept. For instance, if the target concept involves understanding persuasive writing, feedback will focus on the critical aspects such as the development of a clear thesis, the use of supporting evidence, and the effectiveness of the argumentation. This specific, targeted feedback helps students identify and refine the essential parts of their writing, ultimately leading to a deeper and more comprehensive understanding of the target concept. By aligning feedback with the essential characteristics of the target concept, AI technologies can provide more meaningful and constructive guidance, enhancing the learning process and promoting students’ growing understanding.

#### DP2 Learner-Centred Framework Construction

For a writing process to effectively support students’ development and their understanding of the writing process, it should be organised according to the third type of orientation: complete and constructed by students themselves using the provided framework.

#### DP3 Process Visibility

To enhance students’ understanding of the writing process, the overview of the entire process - *operational scheme of thinking*, should be integrated into AI. This integration provides a clear framework that guides students through each step of the writing process, allowing them to see the broader context and purpose of their activities, thereby fostering a deeper understanding of their writing process and the role of feedback in it.

#### DP4 Tangible Feedback Resources

The phase of materialised action suggests that resources (feedback) designed to assist the development of students’ conceptual understanding should be presented in tangible forms, such as texts on web pages, links to relevant resources, and other multimedia formats. These materialised resources provide concrete experiences for students, which are then internalised through collaborative interactions. These interactions facilitate the transfer of materialised actions to the internal plane of the learners, where communicated thinking evolves into dialogical thinking, ultimately leading to mental actions and deeper cognitive engagement.

#### DP5 Collaborative Interaction Spaces

The phase of communicated thinking emphasises the importance of creating opportunities for social interactions during the writing process. These interactions provide valuable opportunities for students to collectively build their understanding of the received feedback and ways of implementing it to improve their texts, thus fostering a deeper engagement in the writing process and enhancing the learning process through social collaboration.

#### DP6 Reflective Dialogical Engagement

The phase of dialogical thinking indicates the need to create spaces withing AI environments for students to express their understanding by creating and/or improving their texts while interacting with the feedback received from AI and discussed with the teacher and the other students. These are the spaces where students can edit, extend and improve their texts by reflecting on the AI feedback.

#### DP7 Integrated Teacher Facilitation

The design of AI learning environments must account for the crucial roles of teacher facilitation in the writing process. Teacher feedback provided to students writing their texts in AI environments can significantly aid in developing their conceptual understanding of feedback, target assignment and assessment criteria while enhancing their learning strategies. This feedback is particularly valued by students during the phases of materialised action and communicated thinking (Engeness, 2018, 2020). In later phases of the writing process, such as dialogical thinking, feedback may be offered upon request or based on the students’ mastery of the writing process they are engaged in (Gamlem & Munthe, 2014; Moltudal et al., 2025).

In summary, these DPs aim to (a) enhance students’ learning in the writing process through their interactions with feedback as a cultural tool, gradually transforming this tool into a meaningful sign; (b) foster students’ conceptual understanding of feedback by revealing the practices of interaction with it and its role in the writing process and (c) develop students’ understanding of the writing/learning process, thus enhance their capacities in learning to learn. By engaging in a writing process in such AI environments, students might improve their learning capacities and become active participants in meaningful knowledge practices. An empirical snapshot is presented to illustrate how these DPs derived from Galperin’s legacy are implemented in the AI-powered Essay Assessment Technology.

## Empirical Snapshot: Essay Assessment Technology (EAT)

EAT was developed in the Research Project *Artificial Intelligence for Assessment for Learning to Improve Learning and Teaching in 21st Century (AI4AfL)* funded by the Norwegian Research Council (2022–2026) through the collaborative efforts of an interdisciplinary team of researchers from Østfold University College and Volda University College, in partnership with the educational technology company Hypatia Learning AS. All participants provided Informed Consent to participate in the study. The Informed Consent was reviewed and approved by Ethics Committee of Østfold University College, ensuring adherence to ethical guidelines. The project aims to develop AI-powered technology to foster AfL approach initiated by Norwegian government back in 2010. The report on the evaluation of implementation of AfL practices in Norway (Hopfenbeck et al., 2013) revealed a gap in understanding and executing AfL practices, highlighting deficiencies in the competencies of teachers, school owners, and teacher educators. This gap has significantly impacted the effectiveness of AfL practices in Norwegian classrooms.

The AI4AfL project seeks to help teachers to implement AfL practices by developing an advanced AI technology (EAT) capable of analysing written texts and providing automatic feedback on grammar, content, structure and organisation of students’ texts. There are the criteria used by Norwegian teachers for assessing students’ written work in English as a Second/Foreign Language (ESL/EFL) classes. The AI technology EAT was developed based on the DPs inspired by cultural-historical theory, presented above. Additionally, the AI4AfL project aims to generate new insights into how teachers organise and facilitate AfL practices using EAT to enhance students’ learning-to-learn capabilities. In doing so, the project aims to bridge theory and practice by offering valuable perspectives to teachers, school owners, and teacher educators on how AI-supported AfL practices can aid in developing students’ understanding of learning in ESL/EFL writing classes.

**Fig. 1:**
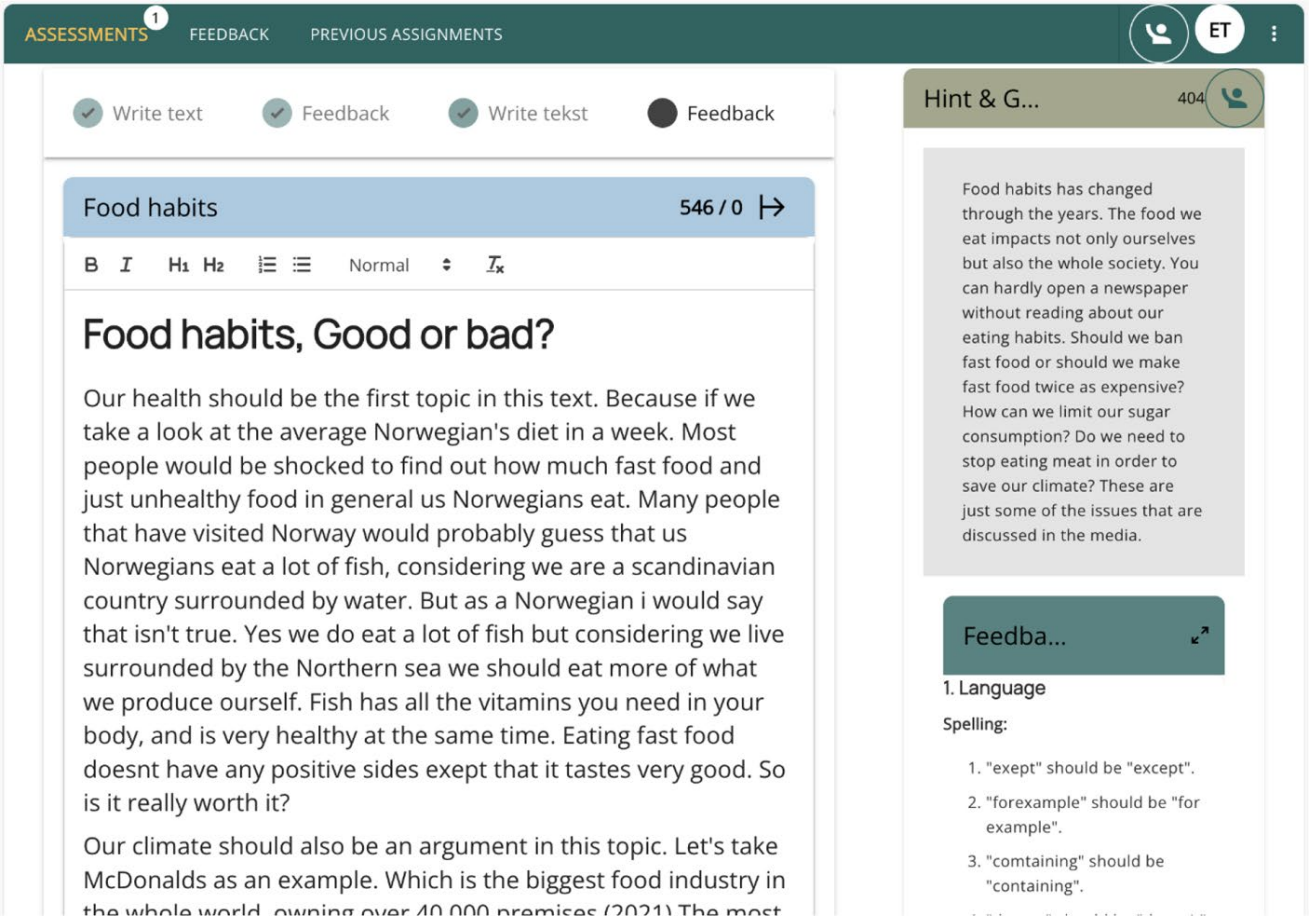
Screenshot of EAT (student view)

The designed AI technology EAT is intended to support the following writing process:

In *Step 1*, the students were to produce their first drafts on the target assignment (in the first two rounds of data collections, the students were to write an essay on the topic Hero (in the first round) and Food Habits (in the second round). The length of their essays was about 400 words. In *Step 2*, the students received individually tailored feedback from EAT which they discussed in the groups of four. The main purpose for these discussions was to create premises for the students to develop their understanding of the received feedback and help each other to apply the feedback in the improved drafts of their essays. The students’ discussions initiated by the feedback from EAT reflected the phases of materialised action (students’ interactions with the feedback) and communicated thinking (students’ meaning making of the feedback during group discussions). Teacher facilitating and guidance while addressing students’ needs was crucial during group discussions. In *Step 3*, the students were to develop their second drafts while reflecting on the feedback from EAT, group discussions and teacher guidance. This step reflected the phase of dialogical thinking when the students internalised the experience they developed in previous phases while creating dialogue with themselves as another person during their writing. *Steps 4* and *5* repeated students’ actions in *Steps 2* and *3* and the third draft created in *Step 5* was submitted to the classroom teacher for evaluation. Figure 1 presents a screenshot of the AI-powered EAT developed in the AI4AfL project:

In the student view, the text of the assignment was integrated in EAT and was constantly visible for the students reflecting the Pedagogical Design Principle 1 (DP1). For example, during this round of data collection, the students were to write a five-paragraph essay about Food Habits. Though engaging in this assignment, the students were to develop their understanding of the concept of Food Habit and the concept of a five-paragraph essay. The text of the assignment reflected the main characteristic features of both concepts:

*Food habits have changed through years. The food we eat impacts not only ourselves but also the whole society. You can hardly open a newspaper without reading about our eating habits. Should we ban fast food*,* or should we make fast food twice as expensive? How can we limit our sugar consumption? Do we need to stop eating meat in order to save our climate? These are just some of the issues that are discussing in the media.*

*Write a five-paragraph essay about people’s food habits and consequences they may have. Present some positive and negative sides of different food habits. Write a conclusion in your essay. You may include the following effects of different food habits on our climate*,* health*,* economy*,* animal welfare*,* customs and traditions and other relevant arguments.*

## Use this Checklist as a Guide before you hand in your text

### • I have Written Five Paragraphs

#### • I have a Suitable Headline

##### • I have Mentioned some pros – positive Aspects pf Various food Habits

- *I have mentioned some cons – negative aspects of various food habits*.

### • I have Written a Conclusion

**Fig. 2:**
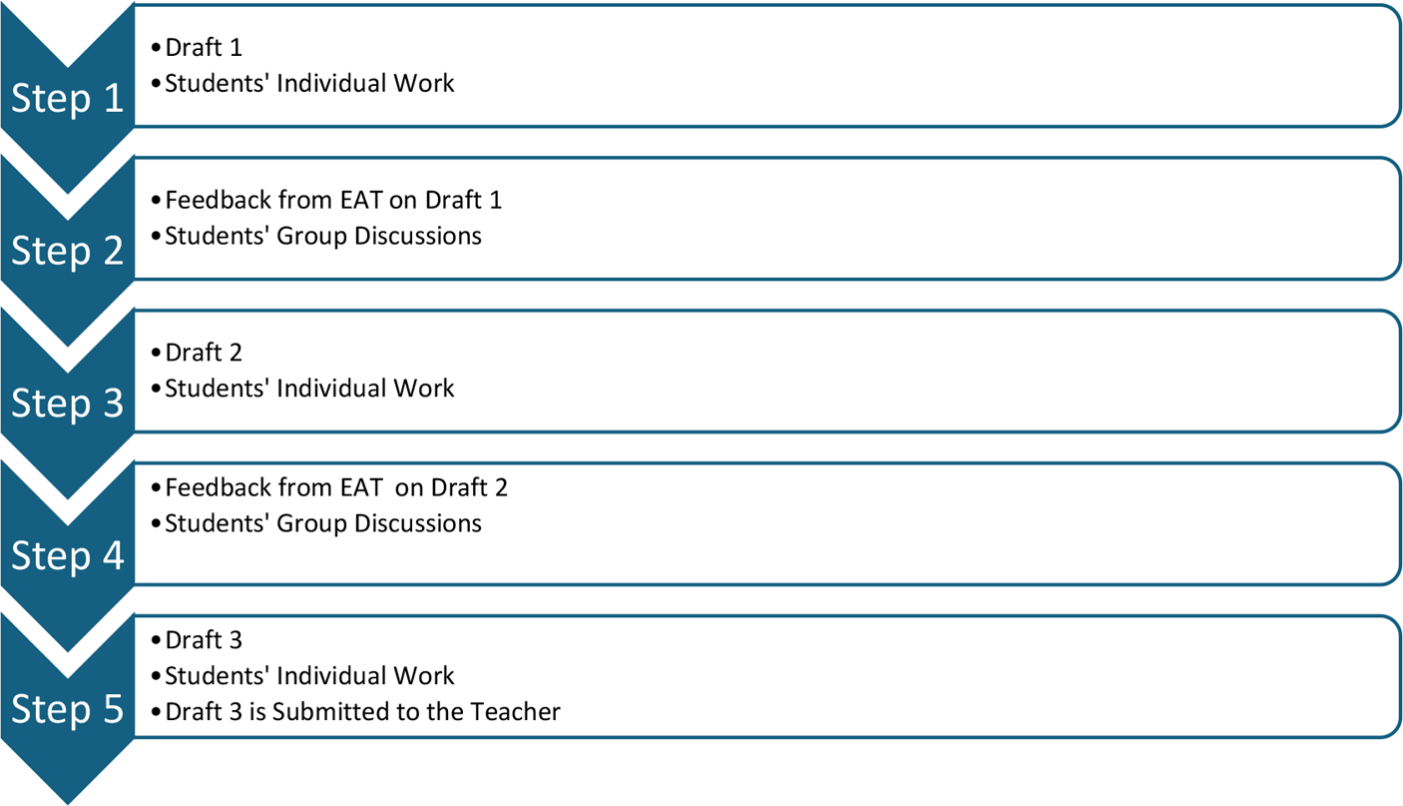
Students’ Writing Process with the Feedback from EAT

The writing process was organised according to the third type of orientation (DP2), providing a framework applicable for writing different types of texts on various topics (Fig. 2). The entire process was visible to the students (DP3 – creating an operational scheme of thinking), enabling them to track their progress and identify their current stage in the writing process. The feedback was presented for the students next to their texts which allowed the students to make connections between the feedback and the text that could be improved. The feedback was presented as a materialised object used to initiate group discussions (DP4) and organised in distinct categories allowing students to develop their conceptual understanding of the feedback on Language, Content, Structure and Organisation of their texts. For example, the feedback on Language was further categorised into spelling, punctuation and grammar; and the feedback on Content was categorised into covered subthemes and suggested subthemes (DP1). The phase of communicated thinking (DP5) was integrated in EAT during students’ group discussions to make meaning of the feedback they received, and the phase of dialogical thinking (DP6) was reflected in Steps 3 and 5 where students created their second and third drafts following the group discussions and interactions with the teacher. DP7 was particularly reflected in the teacher view of EAT that allowed tracing the development of students’ texts in real time and addressing students’ needs. Further, the feedback offered for the students could be summarised and categorised for the teachers indicating the issues that should be followed up with the students.

In summary, the AI-powered technology EAT embodies DPs inspired by the cultural-historical theory. By developing this technology, we offer an approach how to bridge theoretical frameworks with practice to enhance students’ learning. Feedback provided by AI-driven EAT is used by students as a cultural tool that mediates their learning. Integrating the entire writing process within EAT enhances students’ writing experiences and reinforces the Assessment for Learning (AfL) approach, ultimately promoting more effective and self-regulated learning environments.

## Concluding Reflections

While the discussion on the opportunities and challenges of using AI-driven technology for educational purposes and more specifically to support students’ writing process and enhance AfL practices, is timely and important, it seems crucial to bring solid theoretical grounds to develop our understanding of the pedagogical potential of the emerging AI technologies. The mediational role of the cultural resources for learning and development of humans is central to the cultural-historical theory (Lund & Engeness, 2020). This theory, and particularly Galperin’s contribution (Galperin, 2023; Galperin, 1966, 1968), provides a valuable framework for conceptualising feedback as a cultural tool, highlighting its pedagogical potential when embedded within a carefully designed writing process. Within this approach, feedback on students’ drafts acquires a guiding function—not as a mere reflection on completed work, but as a dynamic resource that not only actively supports students in developing their essays to meet the assessment criteria established by teachers but also indicates how they should engage with the writing process. This innovative understanding of feedback extends and transforms traditional conceptualisations, which often positioned it as a post hoc pedagogical tool aimed at fostering reflection on completed work (Black & Wiliam, 2009; Hattie & Timperley, 2007). With the advent of AI technologies, feedback can now be offered on even the smallest units of text, such as a single sentence. By doing so, it acquires its guiding function, enabling students to develop their texts in alignment with learning aims and assessment criteria. To initiate and facilitate productive interactions between students and teachers with feedback, the DPs for AI-powered educational technologies were proposed to support students’ writing processes and enhance the AfL approach. The AI technology EAT designed after these DPs can be considered an *artifact* – a human-made object that provides means of understanding the activities learners engage in (Kaptelinin & Nardi, 2012; Miettinen & Paavola, 2018). To conclude, the discussion in this study can be summarised in the several points.

*First*, when considering using AI technologies to offer feedback in the writing process, it is important to evaluate the mediational and pedagogical potential of the feedback offered by the AI. Our discussion on feedback as a cultural tool might offer useful insights to develop such an understanding. This study discusses how EAT may exemplify the pedagogical role of feedback transforming it from a static assessment to a dynamic learning resource.

*Second*, feedback, as a cultural tool, aimed to guide and improve students’ learning is closely connected to and is influenced by the design of the learning process. The DPs for developing AI-powered pedagogical technologies to support student writing process might be insightful not only for creating new and improving existing AI technologies, but also for designing of writing processes in classrooms. In summary, these DPs aim to help learners to reveal the pedagogical potential of feedback and enhance their understanding of the writing process.

*Third*, the AI-powered technology EAT, designed based on the suggested DPs, can help students not only recognise the potential of feedback for improving the quality of their texts but also navigate the writing process more effectively. This approach enhances students’ understanding of the writing process while enabling EAT to serve a new function—*as a tool for studying the essence of writing*.

*Finally*, although new AI-powered technologies emerging with incredible speed might seem promising for educational purposes, established learning theories might be useful to evaluate the pedagogical potential of these technologies. In our study, it is argued that the cultural-historical theory and, in particular, Galperin’s legacy might offer valuable epistemological grounds for understanding the pedagogical potential of AI-powered technologies to guide their responsible and effective integration into educational practices.

## Data Availability

No datasets were generated or analysed during the current study.
